# The Voynich manuscript: Symbol roles revisited

**DOI:** 10.1371/journal.pone.0260948

**Published:** 2022-01-27

**Authors:** Vladimír Matlach, Barbora Anna Janečková, Daniel Dostál

**Affiliations:** 1 Department of General Linguistics, Palacký University in Olomouc, Olomouc, Czech Republic; 2 Department of Psychology, Palacký University in Olomouc, Olomouc, Czech Republic; University of Sao Paulo, BRAZIL

## Abstract

In this article, we employ simple descriptive methods in order to explore the peculiar behavior of the symbols in the Voynich Manuscript. Such an analysis reveals a group of symbols which are further analyzed for the possibility of being compounds (or ligatures), using a specifically developed method. The results suggest the possibility that the alphabet of the manuscript is a lot smaller, and steganographic type of encoding is proposed to explain the newly revealed properties.

## Introduction

The Voynich manuscript is a historic document, which comprises 102 folios organized into 20 quires, each containing a certain amount of hand-written text of an unknown writing system and illustrations of a seemingly bizarre nature. The text of the manuscript is hand-written and extends throughout the whole work. Overall, the manuscript contains 37,919 words (with 8,114 word types) separated by whitespace [1]; the body of the text is left-aligned. The text is well structured as can be observed from the combinatorics of the characters and the words. The writing system uses various characters, whose count has not yet been established but varies in a range from 23 to 40 [2]. While some characters are simple and could be written by a simple stroke, several are more complicated–those are conventionally called *gallows*.

So far, the research of the manuscript has revolved around three dominant theories about the nature of the text. The first of them is the claim that *it is a plaintext of a natural language*. This conclusion has been made by multiple scholars, e.g. [3, 4], and is usually based on the comparison of the manuscript to other historical codices. The second dominant theory is that *the manuscript is a hoax*–an elaborate forgery made with the vision of monetary gain. From the history of the manuscript, we learn that it was bought by emperor Rudolph II for a large sum of money. For more about the hoax theory [5, 6]. The last, third, dominant assumption is *the ciphertext theory*. This one treats the manuscript as an elaborate historical cipher, which has not yet been decrypted; some encryption methods have been proposed [7].

The history of the manuscript is quite uncertain. It is named after Wilfrid Voynich, a bookseller who found the manuscript in Italy in 1912. Based on a letter which was folded into the manuscript, we can trace back its history to the turn of the 16^th^ and 17^th^ centuries’ Prague, where it was in possession of emperor Rudolph II, who was well-known for his interest in alchemy and occult matters. Even though the place of origin of the manuscript and its earliest whereabouts are not known, thanks to the carbon-dating method, an approximate date of its creation has been established: 1404–1438 (more details in [8]).

**Fig 1:**
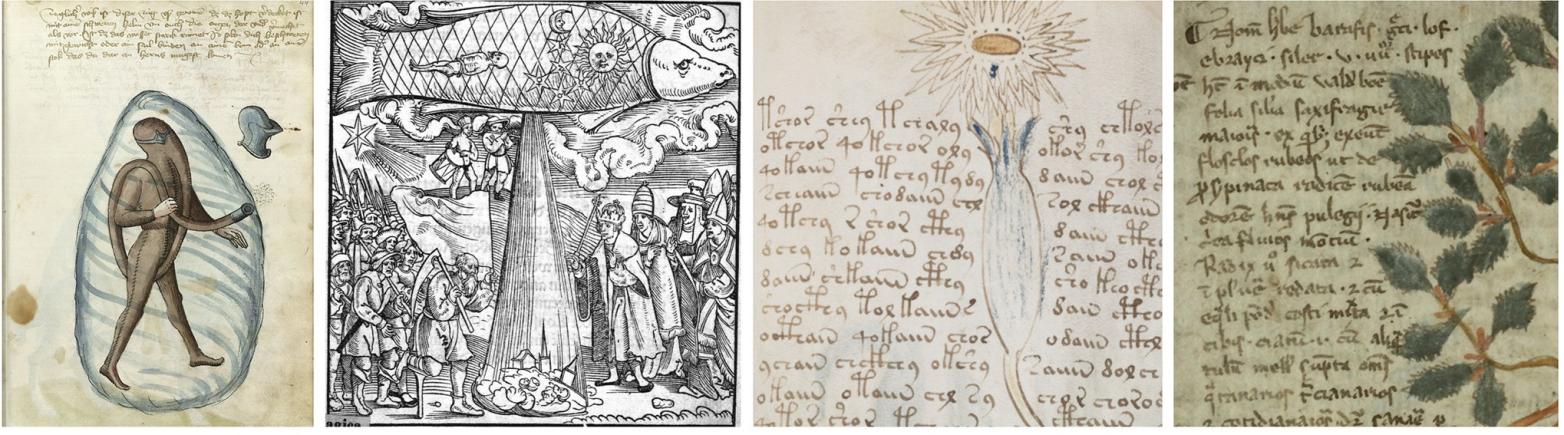
Four examples of historical codices. From left to right: *MS Thott*.*290*.*2°* (1459; Photo: Royal Danish Library); *Practica uber die grossen und manigfeltigen Coniunction der Planeten* (1523; Photo: SLUB Dresden); *Voynich Manuscript* (Photo: Beinecke Rare Book and Manuscript Library, Yale University); *MS O*.*2*.*48* (14^th^ century; Photo: Trinity College Library, Cambridge). Just the Voynich Manuscript is considered as mysterious. (Selected by authors of this article by their discretion).

**Fig 2:**
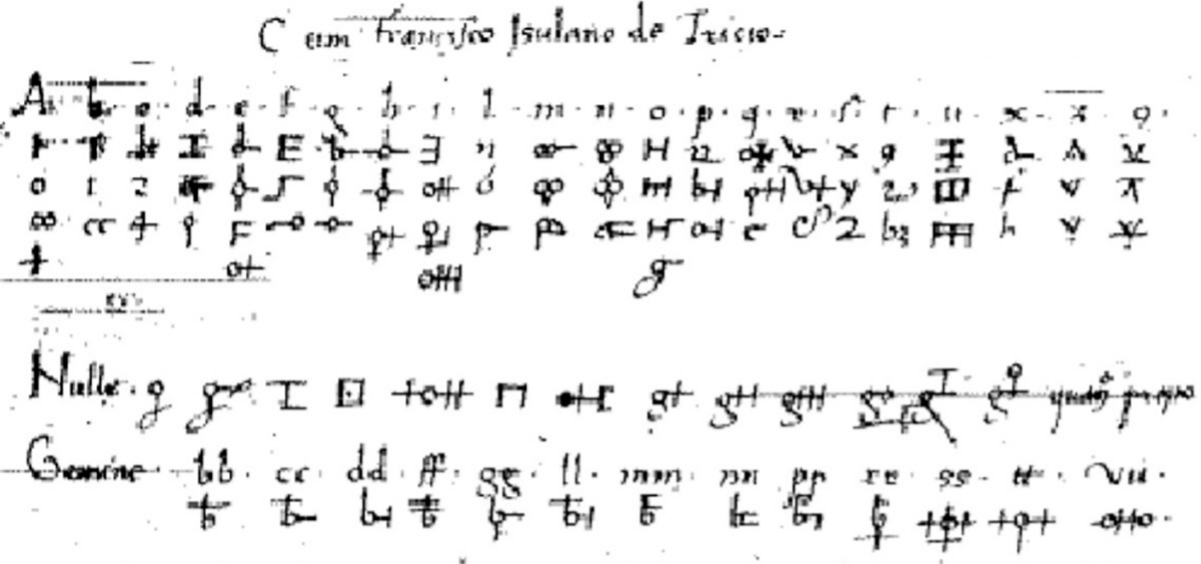
Homophonic alphabet example. *Franciscus Tranchedinus*, Codex *Furtivae litterarum notae*. Vindobonensis 2398, ca. 1450, showing the practice of using artificial, homophonic alphabet to conceal messages. Photo: Augusto Buonafalce (2008).

Given the bizarreness of the manuscript, it is a good idea–as Knight [2] points out–to *adjust* our perception by comparing it to contemporary or newer codices, see Fig 1 for some examples; doing so, the Voynich manuscript loses a bit of its mystique. Also, encryption of manuscripts, or even books, was not out of the ordinary (see e.g. a survey in [2]). The knowledge of breaking substitution ciphers had been known for four centuries [9] in the proposed time of creation of the manuscript. New encryption methods, such as adding homophony and using newly created symbols, see Fig 2, were also introduced in the time. What makes the manuscript so extraordinary is the unknown nature of the contained code and the overwhelming text length, which has not helped with the text decoding in any way.

Up to this day, a staggering number of qualitative and quantitative methods has been applied in order to gain insight into the manuscript. Regarding the quantitative methods, first, well-established linguistic empirical laws were tested, e.g. the presence of the famous Zipf’s law, to understand whether the manuscript possesses assumed fundamental natural text properties (however, as studied, the law itself is a matter of dispute, see [10, 11]. Apart from that, many specific methods were applied–for example calculating unigram word entropy [1], character entropy [12], sequence spectral and correlation analysis [13], or random walk mapping [14] among a plethora of others. We have chosen the path of simple quantitative methods as we view them as providing a great amount of insightful information. Before we step into the analysis of the Voynich Manuscript itself and its symbols, we first formalize the preprocessing of its transliteration by Takeshi Takahashi in 1999, which we used for the analysis and which we found to be the most thorough; see Table 1 for the details. Words containing the illegible symbol placeholder “w” were excluded from the alphabet analysis.

**Table 1:** Text preprocessing table for the Takeshi Takahashi’s transliteration of the Voynich manuscript.

Regular Expression	Replaced by	Comment
[., = %-]+	<space>	White space signaling.
!	/remove/	Extended line information.
*	“w”	Illegible character–removed for position tests; kept for word lengths tests.

Symbols “!”, “*”, “-” were tested and included in both *remove* or *space* groups without any important impact on further analysis.

Furthermore, in order to compare the Voynich Manuscript with various natural languages (which will be conducted later), we preprocess their text samples in the following way: non-alpha characters, except spacing and new lines, were deleted, the texts were then reduced to the first 229,431 graphemes (including the spaces) to be the same length as the processed version of the manuscript. Also, when referring to Voynich text and symbols, we use EVA transliteration signalized by chevrons, e.g. <d> stands for symbol similar to digit ‘8’, for figures we use EVA Hand 1 font (Copyright G. Landini, 1998).

## Periodicity of symbols

First, we examine the repetition (or reuse) of individual symbols. Repetition of symbols can be viewed as a time-series problem analyzing the *delays* between reuse of a given specific symbol. To illustrate this, consider a simple text “XXyXyyXyyyXXyXyyXyyyX” with an examined symbol “X”. Calculating the delays between the reuse of this symbol yields a time-series <1, 2, 3, 4, 1, 2, 3>, which is a very obvious pattern, yet not so simple to spot in the text.

One of the simplest yet effective methods for revealing such patterns is an Autocorrelation Function (or ACF). ACF calculates Pearson correlation coefficient of a given time-series and of its own delayed (*lagged*) version by *n* steps. The process thus quantifies the dependency between current state and the state of *n* time steps in the past. The *n* also iterates from lag 1 until the maximal number of *K* lags (which is calculated specifically for the time-series length) to provide a broader view. The result for one lag varies in a range of -1 to +1, where 0 means no observed correlation (dependency) at all, and +-1 means ideal correlation (dependency). In reality we do not expect correlations as ideal as +-1; however, if they at least surpass a statistically calculated threshold, they can be considered as significant and being caused by some sort of rules rather than by random. Such thresholds are 95% asymptotic confidence intervals described in [15]; for more on ACF itself, see [16].

**Fig 3:**
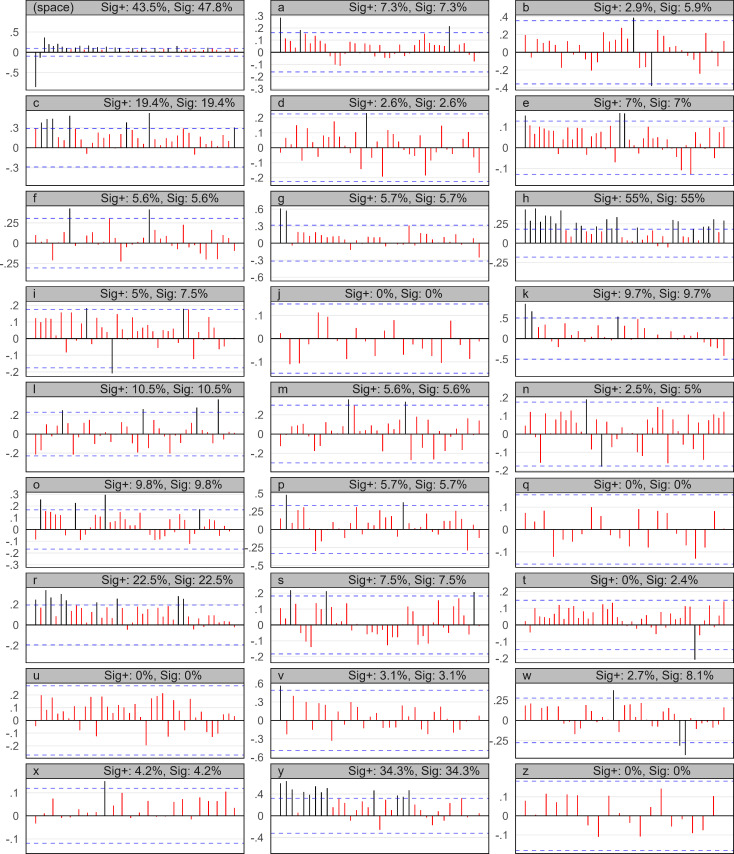
Autocorrelation plots for an English text and its individual symbols. Symbols with a frequency > 100 are shown. The *x* axis shows lags, starting at 1, from left-to-right; *y* axis shows the Pearson correlation; blue horizontal lines are the statistical significance levels. ‘Sig+’ stands for the percentage of ‘significant and positive’ lags, ‘Sig’ stands for ‘significant’ lags. Expected chaotical behavior of symbol repetition may be observed.

Individual symbols in natural languages are not usually expected to *wait* for their reappearance in dependence on the last delay they waited to reappear (except for perhaps when sticking to a specific topic or due to esthetics i.e. in poetry). For illustration, we examine such *waiting* behavior of all individual symbols that occurred at least 10× in an example of an English fiction book. The resulting autocorrelation plots, showing the 27 symbols and their 40 tested lags, can be seen in Fig 3. The *x* axis of the individual plots signalizes the lag, the *y* axis signalizes the assessed correlation coefficient, and the horizontal blue lines demark the estimated statistical significance levels (that need to be exceeded not to reject the rule-based nature of the repetition at the given lag). Although we may find some statistically significant dependencies for some of the symbols and some of their lags, most of them, approximately 88% as we see in summarization Table 2, are not significant.

**Table 2:** Summarization of ACF analysis of individual symbols of a single English text.

Symbol	Sig, ACF [%]	Sig, +ACF [%]
a	7.3	7.3
b	5.9	2.9
c	19.4	19.4
d	2.6	2.6
e	7.0	7.0
f	5.6	5.6
g	5.7	5.7
h	55.0	55.0
i	7.5	5.0
j	0.0	0.0
k	9.7	9.7
l	10.5	10.5
m	5.6	5.6
n	5.0	2.5
o	9.8	9.8
p	5.7	5.7
q	0.0	0.0
r	22.5	22.5
s	7.5	7.5
t	2.4	0.0
u	0.0	0.0
v	3.1	3.1
w	8.1	2.7
x	4.2	4.2
y	34.3	32.3
z	0.0	0.0
[space]	47.8	43.5
Mean	11.9	11.0

The results show approximately 11.9% of all 961 tested lags passed the significance threshold, while 11.03% of the 961 lags are both significantly and positively autocorrelated.

**Fig 4:**
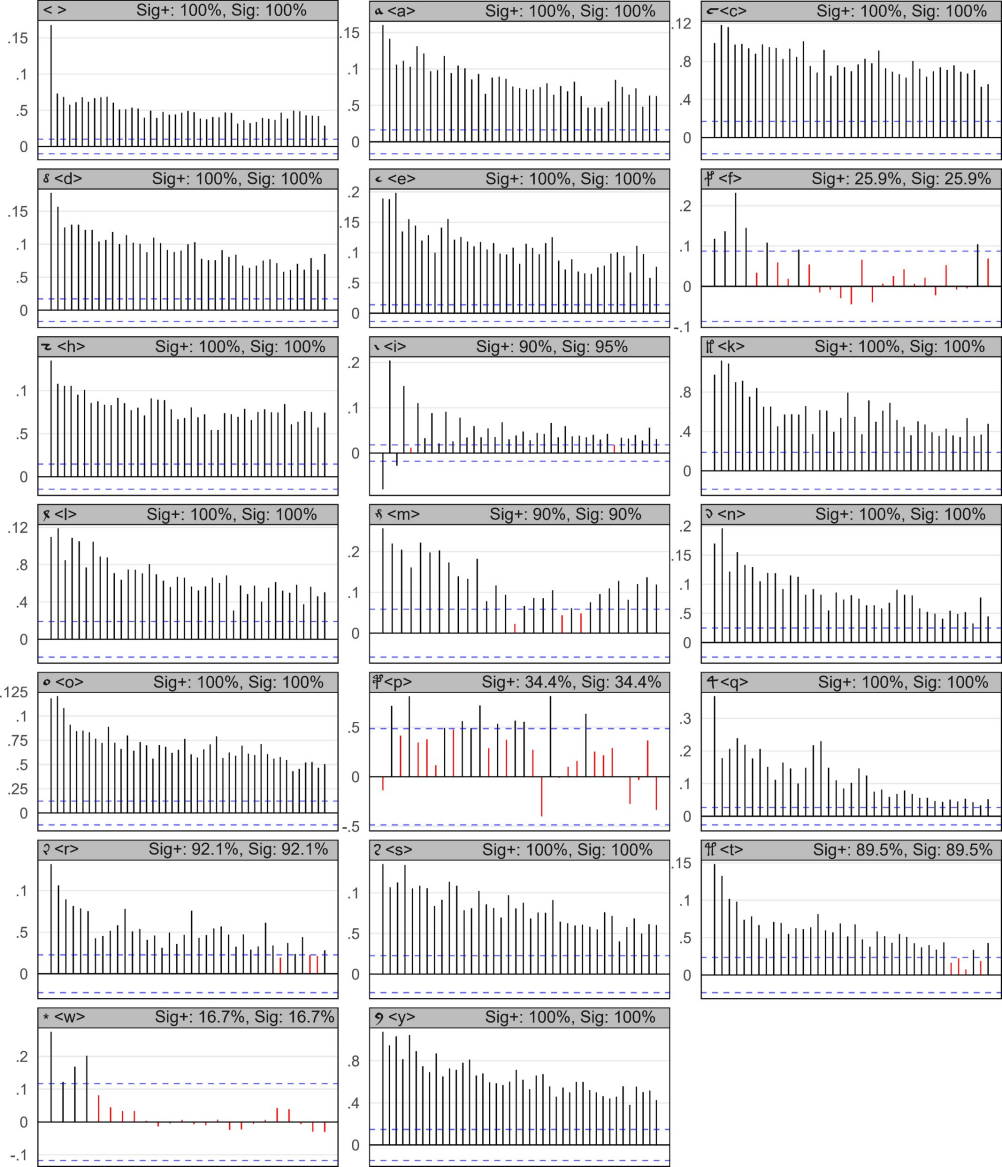
Autocorrelation plots for the Voynich manuscript and its individual symbols. Symbols with a frequency > 100, analogical to the previous Fig 3. The *x* axis shows lags, starting at 1, from left-to-right; *y* axis shows the Pearson correlation; blue horizontal lines are the statistical significance levels. ‘Sig+’ stands for the percentage of ‘significant and positive’ lags, ‘Sig’ stands for ‘significant’ lags. Unexpected behavior of symbol repetition may be observed.

Next, we examine the Voynich manuscript in the same way: For each symbol that occurred at least 100×, ACFs were computed and plotted. The results in Fig 4 show significant differences from the previous English text example. In this case, the majority of the symbols shows significant autocorrelations for all the 40 tested lags. As shown in summary Table 3, 90.4% of the 760 tested lags are significant, while 90.1% of the tested lags are both significant and positive. This observation reveals the presence of autoregressive behavior in nearly all the individual symbols. In other words, nearly all the symbols reappear after delays that are dependent on the previous delay lengths. We may also notice that some symbols, namely <f, p>, behave chaotically as a regular English letter (which is quite peculiar in the context), that symbols <i, m, t, r> have some regularity flaws. A specific behavior is shown by <i>, spiking at each second lag (even/odd) up to (approximately) lag 20, and by <r>, spiking at lags 1, 11, 20 and 30. We will return to these misbehaving symbols later. The space and thus the word lengths are also positively and significantly autocorrelated, implying a possible dependency of the current word length on the previous word lengths (at least up to 40 tested legs).

**Table 3:** Summarization of ACF analysis of individual symbols of the Voynich manuscript.

<EVA>	Sig. ACF [%]	Sig. +ACF [%]
*	16.7	16.7
f	25.9	25.9
p	34.4	34.4
t	89.5	89.5
m	90.0	90.0
r	92.1	92.1
i	95.0	90.0
a	100	100
c	100	100
d	100	100
e	100	100
h	100	100
k	100	100
l	100	100
n	100	100
o	100	100
q	100	100
s	100	100
y	100	100
[space]	100	100
Mean	90.40	90.13

The results show that approximately 90.4% of all 760 tested lags passed the significance threshold, not rejecting the possibility of existing rules determining delays between reusing nearly all the symbols. Also, 90.1% of the 760 tested lags are significantly positive.

Such unusual auto correlations, pointing to the possible presence of an existing system or a memory in the process of the text creation, were also observed on various levels by many scholars: by Landini [13] on the whole manuscript sequence by using equal-symbol multiplication; by Schinner [14] on translation of the manuscript into binary sequence; by Montemurro and Zanette [17] on word use; by Vonfeld [18] on all-symbol-position sequence; and by Arutyunov et al. [19] when assessing Hurst exponent on predefined symbol repetition delays. However, such an analysis–to our knowledge–has not yet been done on all individual symbols. Next, we use this presumably uncommon text behavior for comparison with other languages and text samples.

Comparing ACF results of 28 various text samples–see the whole list in Table 4 and S1 Table–and listing all their symbols would be extremely space-consuming. Therefore, we summarize each tested language sample into a set of its descriptive ACF measurements: *mean*, *median*, *standard deviation* (SD) and its *quantiles* Q_2.5%_ and Q_97.5%_. The samples were also manually assigned into groups for aiding perception of the following plots and possible clusters.

**Table 4:** Description of ACF values distributions of all contained symbols for various language samples.

Language sample	Group	Mean	Median	SD	Q_2.5%_	Q_97.5%_
Voynich	Voy.	.073	.068	.044	-.009	.189
Voynich generator [20]	Voy.	.070	.068	.054	-.031	.193
Bengalian (Bible)	B	.044	.037	.047	-.035	.160
Korean (Bible; hangul)	C	.044	.033	.079	-.087	.235
Arabic (Bible)	A	.037	.033	.040	-.035	.129
Russian (fiction w/ passages in French)	D	.034	.008	.063	-.030	.217
Armenian (Bible)	A	.029	.024	.033	-.028	.116
Kannada (Bible)	B	.024	.018	.039	-.045	.111
Latin (Bible)	A	.024	.018	.031	-.016	.097
Chinese (mix traditional and new)	C	.023	.012	.078	-.107	.215
Hebrew (Bible)	A	.020	.016	.027	-.020	.072
Mokole (Bible)	A	.019	.015	.028	-.033	.088
Eskimo (Bible)	B	.016	.011	.035	-.034	.088
Rigveda (hymns in Devanagari)	D	.015	.014	.046	-.076	.121
Polish (Chess Manual)	A	.013	.008	.028	-.033	.081
Czech (fiction)	A	.011	.009	.025	-.028	.066
Greek (fiction)	A	.010	.008	.021	-.030	.051
Italian (Calabrian: Neapolitan, fiction)	A	.009	.008	.024	-.034	.065
English (fiction)	A	.007	.007	.026	-.043	.072
Italian (fiction)	A	.007	.006	.025	-.040	.069
Greek (Homer)	A	.006	.005	.021	-.032	.049
English (Encrypt: Playfare)	Enc.	.006	.006	.012	-.017	.035
Latin (fiction)	A	.004	.002	.022	-.036	.056
Latin (Virgil; 1st Century BCE)	A	.003	.003	.017	-.029	.038
English (Encrypt: Vigenere)	Enc.	.003	.003	.014	-.024	.032
English (Encrypt: Fialka)	Enc.	.000	.000	.012	-.021	.023
English (Encrypt: GPG)	Enc.	.000	.000	.011	-.022	.022

Text samples also includes Voynich Manuscript, its imitation by Timm and Schinner [20] software, texts encrypted by various cryptographic algorithms (demanding or not a source of entropy). The purpose of sample “group” is to simplify the perception of the plots, the groups are assigned manually to highlight potential clusters.

The text samples contain the Voynich manuscript, a Voynich-manuscript-like text generated by software presented in Timm and Schinner [20], 19 various languages, and four samples of different encryptions of a single English text, encrypted by Playfare, Vigenere, Fialka, and GPG methods. The resulting Table 4 is sorted by mean ACF value in descending order. By assessing the results, we may notice the following important information:

1. The Voynich Manuscript has the most extreme mean ACF value, even when compared to the various translations of the Bible containing repeating structures.
2. The most similar text to the Voynich Manuscript (from the perspective of the mean ACF) is the artificially generated text by Timm and Schinner [20] software.

**Fig 5:**
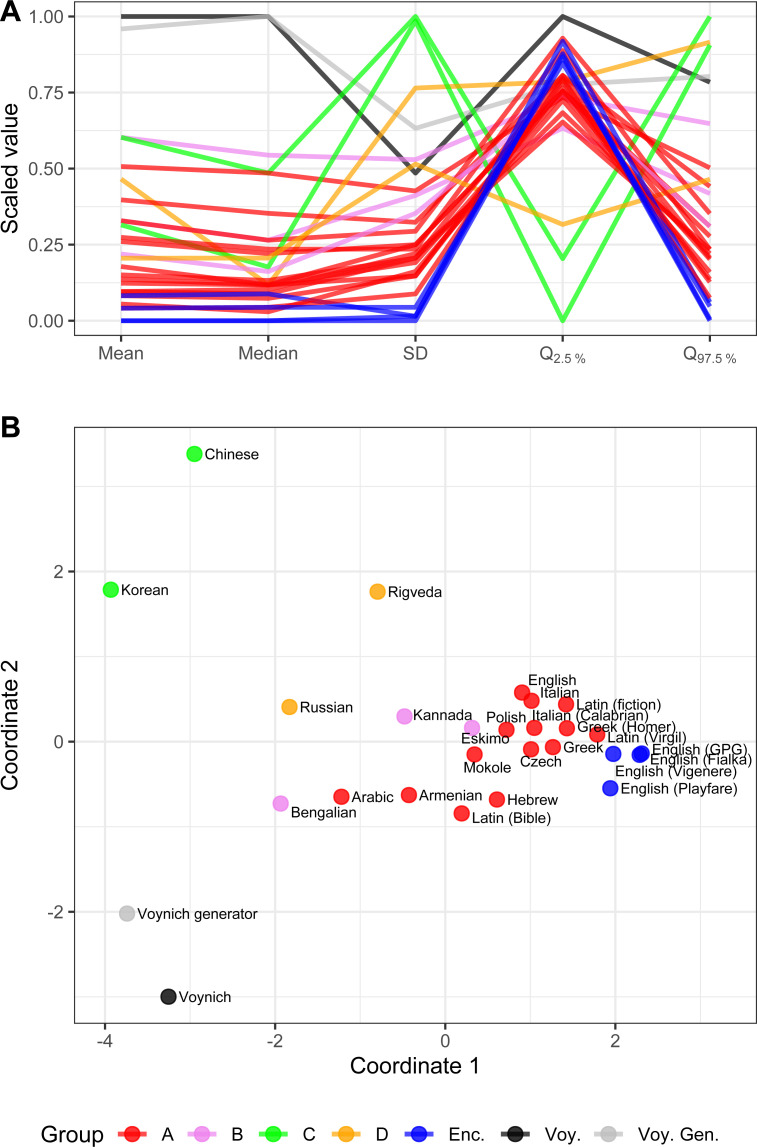
ACF summarization data visualization. Parallel coordinate plot (chart A) and multidimensional scaling plot (MDS; chart B) of Symbol repetition delays ACF summarization data for various language and text samples listed in Table 4. Data were scaled by *min-max* scaling and Euclidean distances were used for plotting the MDS. MDS explains 96.8% of the original variance. Voynich Manuscript along with the Voynich Manuscript imitation are dissimilar to the rest of the tested texts, create anti-patterns (visible in chart A) and form its own clusters (visible in chart B).

By visualizing Table 4 in form of a parallel coordinate plot (where each measurement is individually linearly scaled so the minimum value is zero and the maximum value is one–i.e. *min-max scaling*), see Fig 5A, we gain a quick overview on the text differences. The Voynich Manuscript, along with its imitation, shows different behavior in comparison with the other text samples–even with the encrypted texts. By visualizing the Euclidean distances calculated from *min-max scaled* Table 4 using multidimensional scaling which reconstructs the texts pairwise distances onto a 2D plane [21], see Fig 5B, we find that both the Voynich Manuscript and its imitation are creating their own cluster, which is distant from the other texts. In comparison to the natural language samples and encryptions, the Voynich Manuscript is not only an extreme case, but it is a unique text in the way the symbols are written and how the symbols *wait* for their re-emergence. In line with the results and the fact that the studied property is empirically uncommon, we may speculate that the individual Voynich symbols do not have a role of any symbols we have yet seen in terms of any natural language sample or their common encryptions we tested.

The individual examination of the Voynich symbols autocorrelations presented in Fig 4 provides us more information once we assume that its behavior is indeed unique. The ACF reveals that existence of a system that placed most of the symbols into their specific positions with specific delays between them cannot be rejected due to the observed significant autocorrelations. At the same time, and contrarily due to the observed chaotic ACF, we can reject (with a given certainty) that some of the symbols–primarily the gallows symbols–were either excluded or unnoticed by the system, or were added into the text after the system placed the majority of the symbols. What is also worth mentioning is the fact that the simplest symbol <i> also behaves systematically: in an even/odd nature, which makes it unique from the set of Voynich symbols. Such behavior could be caused by a specific role in the given system, or due to being controlled by another system or interfering with another set of rules.

The last observation stemming from a symbol time series analysis by Fourier Discrete Transform reveals another unique Voynich Manuscript property, which is not present in any other tested text sample. Approximately ½ of the top ten most powerful periods of all Voynich alphabet symbols are integers, while the most of them are divisible by 40 and 160. Other examined text samples showed low numbers of integer frequencies (e.g. 12.2% for English sample, 19.9% for Hebrew sample) while none of the pertinent integers were divisible neither by 40 nor 160.

## Detecting the ligatures

From the ACF analysis above, we find that the Voynich Manuscript contains symbols that behave differently from the others while possibly staying aside from an assumed system that places most of the symbols into specific positions in dependence on the previous decisions. Such misbehaving symbols include the so-called gallows symbols <f, p, t> and symbols <m, r>, all of which can be considered as the most graphically complex symbols of the Voynich alphabet. The simplest symbol <i> is also included in this list; however, in a different way. The correspondence of the graphical complexity and the ACF misbehavior rises a question on the relation of these symbols to the others. As for the graphical complexity, the possible explanation we explore further is that such complex and misbehaving symbols may be ligatures–compounds of the other graphically simpler and also ACF well-behaving symbols.

The possibility that some Voynich symbols are *compounds* of more symbols was already discussed by [22], who (apart from a table of more complex compounds as <ckh>) mentions some ideas: for example, that <a> might be composed of <e> and <i>, or that <d> might be a compound of <e> and <l>. However, she also notes that the decomposition of such symbols is quite ambiguous and of a rather speculative nature.

Following the idea, let us assume that some Voynich symbols are indeed ligatures. First, let us define a ligature: A ligature is a graphical composition of at least two immediately succeeding graphemes combined into a new single glyph, e.g. a string *ae* forms ligature *æ*, or a string *et* forms &. From such a definition we may deduce several heuristic rules, which, in combination, may quantify a *ligatureness* score of any given symbol and its possible candidate components for any given text.

In order to define the ligatureness score of Voynich symbols, we shall first formalize used notation. Let us define the set of all Voynich symbols (the Voynich alphabet) as Γ. Let the symbol suspected of being a ligature–or a *ligature candidate*–be named as C and its candidate components as A and B. Following the example above, the symbol & is a ligature candidate C and symbols *e* and *t* are its candidate components A and B (in a left-to-right order). The probability of a symbol *x*∈Γ is *p*(*x*). Transition probabilities of the symbols (i.e. the probability of symbol *x* being immediately followed by symbol *y*, *w*.*r*.*t*. the sum of all the frequencies of the pairs) define an *incidence* matrix M: all symbols Γ are listed in rows and columns (in the same order) while listing their left-to-right succession probability in cell value. The probability of succession of symbol A to symbol B (or B being preceded by A) is equivalent to notation of M_*A*,*B*_. The probability of succession of symbol B to symbol A (or A being preceded by B) is equivalent to notation M_*B*,*A*_. For the complete incidence matrix M, see S1 Fig. (Let us note, for clarity, that in strict sense, the word *probability* should be regarded as *relative frequency;* however, we find it simpler to address these as probabilities despite slight terminology problems.)

The ligatureness score of a ligature candidate symbol and its candidate components (forming a candidate *triplet*) is calculated by a combination of heuristic rules (1–8) that we have deduced for this purpose. Each rule produces a loss score (or a distance). A triplet yielding a zero loss score possesses the ideal ligature-like behavior. Combination of the rules, which will be formalized later, yielding zero score as the result thus means the perfect overall match of the triplet with the assumed ligature-like behavior. For convenience, we have named each rule, defined the type of examined relations, and supplied brief descriptions with rationales and formalizations. Where appropriate, the rules were scaled. (It is also notable that the individual rules are designed to be differentiable for automated optimization purposes).

### 1) Ligature surpass (Relation: Ligature to candidates)

Since the ligature C substitutes two symbols (A and B), the ligature C should not be used more frequently than the most frequent symbol A or B. In other words, ligature C–as a more complex symbol–should not surpass its use in comparison to use of symbols A or B. Formally, the following should hold: *p*(*C*)≤max(*p*(*A*), *p*(*B*)). This is thus definable as the surpassing quantity $r_{1}$:

$$r_{1}=\left\{ \begin{array}{c} p(C)-\max\left(p(A),p(B)\right)\;if\;p(C)>\max\left(p(A),p(B)\right),\\ 0\;otherwise \end{array}\right.$$

which we normalize by maximal symbol probability to produce the final rule loss score ℓ_1_:

$$l_{1}=r_{1}/\underset{s\in \Gamma }{\max}\;p(s).$$

### 2) Naked occurrence (Relation: Candidates)

If a symbol A succeeded by a symbol B creates a ligature C, then symbols A and B should never occur in succession in the text as they should be replaced by the ligature C. This is a mandatory condition (which can, however, be violated by a scribe or transliteration mistakes). The rule can be formalized as the mere probability of encountering succeeding A and B:

$$r_{2}=M_{A,B}$$

and normalized by maximal transition probability:

$$l_{2}=r_{2}/\max\left(M\right).$$

### 3) Left Context set match (Relation: Ligature to the first candidate)

Since C is the ligature of A and B, the symbols preceding C should *match* symbols preceding symbol A. At the first glance, this may be viewed as a simple two set intersection measurement. However, either A or C will probably have different frequencies and will automatically produce larger context for the more frequent symbol (we name the more frequent symbol X) and smaller context for the less frequent symbol (symbol Y). Naturally, the two symbols do not have equal opportunities to realize the same number of symbols in their left context, which is given by the lower frequency of Y. As this should not be penalized, the loss score is based on the cases where Y is preceded by symbols that do not precede the more frequent symbol X. The loss is thus the sum of the probabilities of Y-to-X non-matching symbols, formally:

$$X=\arg\underset{x\in \{A,C\}}{\max}\;p(x),$$

$$Y=\arg\underset{x\in \{A,C\}}{\min}\;p(x),$$

$$l_{3}=\sum_{x\in \Gamma }\lceil M_{x,X}\rceil \cdot M_{x,Y}.$$

### 4) Left context usage pattern match (Relation: Ligature to the first candidate)

Following the logic of the previous rule–if the symbol A is the first component of the ligature candidate symbol C, then even the probability distribution (the usage pattern) of their left contexts should match. We measure this pattern match by Pearson correlation coefficient. As noted above, even here we need to consider the problem of reduced opportunity to realize all possible left contexts due to lower frequency of one of the assessed symbols A/C. Thus, we calculate the correlation of the probabilities only between the symbols that are realized by the less frequent symbol of them both, which we name Y. Formally, first we define a vector of all realized left-context symbols of this symbol Y:

$$\gamma =\{x\in \Gamma :M_{x,Y},>0\}.$$

Next, we define vectors *α* and *β* containing probabilities of these *γ* symbols being in front of symbols A and C:

$$\alpha =\{x\in \gamma :M_{x,A}\}$$

and

$$\beta =\{x\in \gamma :M_{x,C}\}.$$

Lastly, for the two probability vectors *α* and *β*, we calculate the loss measure ℓ_4_ based on Pearson correlation coefficient:

(3)

$$l_{4}=1-\left|\frac{\sum_{i=1}^{n}\left(\alpha_{i}-\bar{\alpha })(\beta_{i}-\bar{\beta }\right)}{\sqrt{\sum_{i=1}^{n}{\left(\alpha_{i}-\bar{\alpha }\right)}^{2}}\sqrt{\sum_{i=1}^{n}{\left(\beta_{i}-\bar{\beta }\right)}^{2}}}\right|$$

where *n* = |*γ*|. In the case of missing left context, formally |*γ*| = 0, we assume ℓ_4_ = 0.

### 5) Rare ligature succession (Relation: Ligature)

The probability of encountering the same two immediately succeeding ligatures CC is as probable as seeing the succession of their components ABAB. Such succession is assumably an event with probability *p*_*CC*_ = *p*(*A*)⋅*p*(*B*)⋅*p*(*A*)⋅*p*(*B*). This, however, only applies to infinitely long and random texts (which will also be discussed further). We may simplify this concept and state: the more frequently we encounter candidate C in succession, the less it could be a ligature. The rule is thus defined as:

$$r_{5}=M_{C,C}$$

while producing the final loss ℓ_5_ by normalization:

$$l_{5}=r_{5}/\max\left(diag(M)\right),$$

where diag(M) is the diagonal of the matrix M.

### 6) Constitution (Relation: Ligature candidates)

Constituting a ligature is more probable for frequent components. In other words, the higher the frequencies of symbols A and B are, the higher the possibility to develop the ligature is. We may restate this in a more rational way: The possibility of forming a ligature depends on its least frequent assumed component A or B, as there would be no reason to form a ligature of an extremely frequent symbol with an extremely low frequent symbol. Thus, we define the loss by means of the least probable component:

$$r_{6}=1-\min\left(p(A),p(B)\right)$$

while producing the final loss ℓ_6_ after normalizing by:

$$l_{6}=r_{6}/\underset{x\in \Gamma }{\max}\;p(x).$$

### 7) Estimated Probability (Relation: Ligature to candidates)

If the probability of encountering symbol A is *p*(*A*) and the probability of encountering symbol B is *p*(*B*), then encountering their succession AB is *p*(*AB*) = *p*(*A*)⋅*p*(*B*). If symbol C is indeed a ligature of AB, then, for an infinitely long random text applies *p*(*C*)≈*p*(*A*)⋅*p*(*B*). In other words, encountering a ligature should be as probable as seeing its components in succession. Since we do not work with infinitely long random texts (where generator does not possess any memory nor rules), we may restate the rule into a weakened condition *p*(*A*)⋅*p*(*B*)≥*p*(*C*). We must also remember, that if C is indeed a ligature of AB, then C also realizes instances of A and B and as such, *p*(*A*) and *p*(*B*) should be corrected by adding the *consumed* probabilities of A and B by ligature C. The loss score ℓ_7_ can be formalized as:

$$l_{7}=\frac{p(C)}{\left(p(A)+p(C))\cdot (p(B)+p(C)\right)}.$$

It is important to note the random-text assumption may be still very strict for natural languages or texts containing symbol cooccurrences and yield higher losses for true ligatures even with the weakened form of this rule. The weight of this rule will be thus reduced.

### 8) Same symbol penalization (Relation: Candidates)

We may assume that forming a ligature of the same two symbols is less probable, thus we penalize this possibility by a loss of 1:

$$l_{8}=1\;if\;A=B,otherwise\;0.$$

(Although we may find examples: *vv* in English transformed into *w* or ss in German could be in some cases rewritten as *ß*, the method would be too biased in finding the same symbol candidate components.)

The last step, the overall ligatureness loss score ℒ for a given triplet of ligature candidate C and its assessed components A and B, is defined as a weighted sum of the individual rule losses 1–8:

$$L_{<C,A,B>}=l_{1}\cdot w_{1}+l_{2}\cdot w_{2}+\ldots +l_{8}\cdot w_{8}=\sum_{i\in \{1,2,\ldots ,8\}}l_{i}\cdot w_{i}$$

where $w_{1,\ldots ,8}\in R_{0}^{+}$ are loss weights. The particular values of the weights were derived theoretically and then manually modified in such a way the obtained loss scores ℒ for the selected true ligatures and their components fell under the 1^st^ percentile of all possible triplet loss scores in examined Latin and Arabic texts. Weight setting fulfilling this condition was then fixed and validated. Validation was assessed on an Armenian text, where ligatures occur naturally, and on Czech and English texts with artificially added ligatures (*pa* and *ea*); the aim was to keep ℒ under the 5^th^ percentile without any further weight or method modifications (leaving the whole idea only one chance due to high possibility of an overfit as non-artificial examples of ligatures are limited). The following weights were selected:

w=[1,100,1,1,100,1,1,10].

Rules Naked Occurrence and Rare Ligature Succession were set as the most important (weights of 100), Same Symbol Penalization as moderately important (weight of 10), and the rest of the rules maintained the same weight (1).

Putting these weights into practice, we get the following results: for a Latin text, a ligature triplet [æ, A, E] resulted in 0.15^th^ percentile (rank 22 out of 14,400 possible tested triplets) and a triplet [œ, O, E] resulted in 0.076^th^ percentile (rank 11 out of 14,400 possible tested triplets); for an Arabic text, a ligature triplet [ا,ل,لا] placed on 0.0248^th^ percentile (rank 10 out of 40,460 possible tested triplets). Concerning validation texts, an Armenian ligature triplet [և,ե,ւ] resulted in 2.11^th^ percentile (rank 1,189 out of 56,316), artificially added English triplet [ý, e, a] yielded 0.2nd percentile (rank 37 out of 18,252) and artificially added Czech triplet [q, p, a] yielded 0.01st percentile (rank 405 out of 40,176).

As mentioned before, even though the rules and the weights are designed for detecting ligatures, we cannot expect the best possible results, namely the right answers to be placed on the first position(s). This is due to the fact that the examined texts are not infinitely long and random. Even natural languages, despite their numerous arbitrary levels, have many regularities (which are possibly stemming from phonotactics or from morphology of a given language) that will negatively affect some of the defined rules above. From the natural language examples, we also noticed that the triplets which placed with the near-ideal loss scores contained either the right ligature candidate or both its right components. As we will see later, also mistakes made by scribers or transliterators negatively affect the final triplet loss scores. For such reason, we also add one more rule–the rule 9 –which is not participating in the loss score anyhow:

### 9) Visual traits

Ligatures should possess visual traits of their components and their manual writing must be senseful. In other words, making a ligature must be visually understandable and writing a ligature should be easier than writing the two components alone.

With all the rules set up, the next step is evaluating ligature loss score ℒ for all possible Voynich symbol triplets. For this analysis, symbols with frequency higher than 500 are used (based on a sudden frequency drop from 503 to 94 and fewer occurrences for the rarest symbols), yielding 18 testable symbols (out of original 23) producing in total 18×17×17 = 5,202 testable triplets (triplets containing the ligature candidate symbol as its own component(s) are discarded due to their production of infinite regressions). Also, for clarity, we name this set of all testable triplets Λ. The ligature test is assessed on the whole manuscript, as the eventual ligatures–as we assume–should be part of the general Voynich writing system and thus should be used independently from the two quantitatively and qualitatively different Voynich *languages* A and B, which were described by Currier [23].

Before we start evaluating the individual loss scores, it is critical to check the incidence matrix M as it is the basis of most of the calculations and its inconsistency would harm the results. This matrix may contain at least two types of mistakes: First, it can reflect wrong transitions stemming from scriber mistakes–such mistakes we can barely prove, and second, wrong transitions stemming from transliteration mistakes, which we can manually verify and which are our primary concern. As we show in several examples of S2 Fig, the suspicion of mistaken transliterations anticipated from low relative transition probabilities in matrix M, has been confirmed (e.g. many symbols <m> may be attributed to <g> after verifying high quality scans). This process of manual verification and following text repairment is, however, truly exhausting. For this reason, we stepped to zeroing all transitions with probabilities less than 0.2% w.r.t. their row and/or their column (e.g. a symbol transition with frequency of 5 will be zeroed if 5 is less than 0.2% of row sum or column sum), formally:

$$M_{x,y}=\left\{ \begin{array}{c} 0\;if\;min\;(M_{x,y}/\sum M_{x,\ldots };M_{x,y}/\sum M_{\ldots ,,y})\times 100<0.2,\\ M_{x,y}\;otherwise. \end{array}\right.$$

Even though the 0.2% is a low threshold, the process zeroes 186 low-probability incidences–for the final form of the incidence matrix M, see S1 Fig. Now, with suspicious transitions removed, we step to evaluating the individual loss scores.

As there would be no room to list all the 5,202 losses for each examined triplet, first, we list the evaluation results for each tested Voynich symbol (as if assumed they are all ligature symbols) with their best components guess. Results include rule losses (shortened as *R*_*n*_ = ℓ_*n*_×*w*_*n*_), ligatureness loss score ℒ, percentage *P* of getting such or a lower (better) ℒ_*T*_ (or formally *P* = *p*(ℒ_*x*_≤ℒ_*T*_) for *x*∈Λ and given triplet T), signalization of previously observed *peculiar* ACF behavior (from Table 3; shortened to p-ACF, which we define as *not having all ACF lags significant*) and an estimate of the ligature symbol graphical complexity GC (derived from the number of pixels needed to write the symbol in EVA Font 1). The results, sorted by the loss score ℒ from the best (lowest) to the worst (largest), are listed in Table 5.

**Table 5:** Enumeration of the best ligature candidates and their components.

<Triplet>	R_1_	R_2_	R_3_	R_4_	R_5_	R_6_	R_7_	R_8_	ℒ	P	GC	p−ACF
f i o	0	0	.001	.070	0	.539	.304	0	.915	.019	348	✓
m o h	0	0	.001	.657	0	.299	.424	0	1.380	.327	254	✓
p i o	0	0	.004	.049	0	.539	.862	0	1.454	.404	396	✓
q o h	0	0	0	0	0	.299	1.443	0	1.742	.865	209	
n o h	0	0	0	0	0	.299	1.550	0	1.848	1.403	208	
r o h	0	0	.019	.546	0	.299	1.712	0	2.577	3.279	223	✓
t o h	0	0	.032	.637	0	.299	1.653	0	2.621	3.845	423	✓
d o h	0	0	.060	.393	0	.299	2.095	0	2.847	4.633	227	
k o h	0	0	.057	.798	0	.299	1.997	0	3.150	5.805	409	
y o h	0	0	.103	.588	0	.299	2.207	0	3.196	5.998	219	
c i o	0	0	.002	.049	0	.539	2.624	0	3.214	6.055	157	
a i o	0	0	.002	.049	0	.539	2.644	0	3.234	6.113	138	
s o h	0	0	.017	.696	.601	.299	1.705	0	3.318	6.478	254	
l o h	0	0	.039	.701	.543	.299	1.972	0	3.554	7.593	168	
h i o	0	0	0	0	1.668	.539	2.666	0	4.873	15.283	137	
o e h	.212	0	.130	.538	1.765	.299	2.471	0	5.414	19.512	138	
i o h	0	0	.002	.049	9.343	.299	2.040	0	92.734	88.639	58	*
e o h	0	0	.130	.538	100	.299	2.224	0	103.191	93.253	93	

R, rule loss scores; ℒ, triplet loss score; P, percentile; GC, graphical complexity; p-ACF, observed peculiar ACF behavior.

The first thing we notice is a tendency of placing the ACF-peculiarly-behaving symbols at the best-scoring positions. All these symbol scores also lie below the 5^th^ percentile of all triplet losses–except the symbol <i>, which also showed a completely different ACF behavior. What can further be observed, based on GC column, is a tendency of placing visually complex symbols at better ligature scoring positions. Graphically simplest symbols are then placed at the bottom of the table with the worst scores. This observation may also be quantified by measuring the correlation of the two properties ℒ and GC. The resulting Spearman rank correlating *r* = −0.731 (p-value = 0.0006) and Pearson Correlation Coefficient *ρ* = −0.52 (p-value = 0.026) both point to statistically significant correlation between the designed loss score and estimated symbol graphical complexity. Assessing the top scoring component guesses, however, shows a certain bias towards symbols <i, o, h>—therefore, as was already stated above, we shall not rely on them. Rather, we shall examine all the offered components of each symbol visually and conduct so-called *greedy search*: We will proceed from top to bottom and accept the first visually plausible option. This will be done for all the Voynich symbols that scored under the 5^th^ percentile.

To second this analysis and to provide ground for identifying the reasons why the other components scored badly, even if they may look more fitting for the symbol as its components, we add two more pieces of information to the individual rule losses. First, we add a *drawback percentage p*_*R*_, which we define as a probability of gaining any lower (better) score for the given rule than we currently observed in comparison to all other triplet scores, i.e. *p*_*R*_ = *p*(*x*<ℓ), where *x* are other triplet scores of the examined rule and ℓ is the examined loss score. Second, we add loss contribution percentage signalizing the relative contribution of each rule to the overall loss score of a given triplet (formally *R*_*i*_/∑*R* for a given rule *i;* note that the stated rule losses are weighted). All of this information is then plotted into a summary chart. For example, a plotted result for a triplet T with R_1_ (on *x* axis) and *p*_*R*_ = 10% (on *y* axis) with point labeled as 0.123 (70%) stands for *the triplet T scored 0*.*123 at Rule 1*, *while there are 10% of better scores for this rule for any other triplets*, *while this (weighted) rule contributes 70% to the overall triplet loss score* ℒ_*T*_.

What should be noted before we move to discussing possible ligatures is the fact that in most cases, the best component guess of the algorithm does not satisfy rule 9, which states that the components of a ligature should be visually plausible. As was mentioned above, even in the case of natural languages which were used to tweak the algorithm, the actual components of the ligatures did not always place first. Therefore, we conduct a greedy search for a pair of components, which satisfy also the last stated rule; for the best component candidates, refer to Table 5.

### Ligature candidate <f>

**Fig 6:**
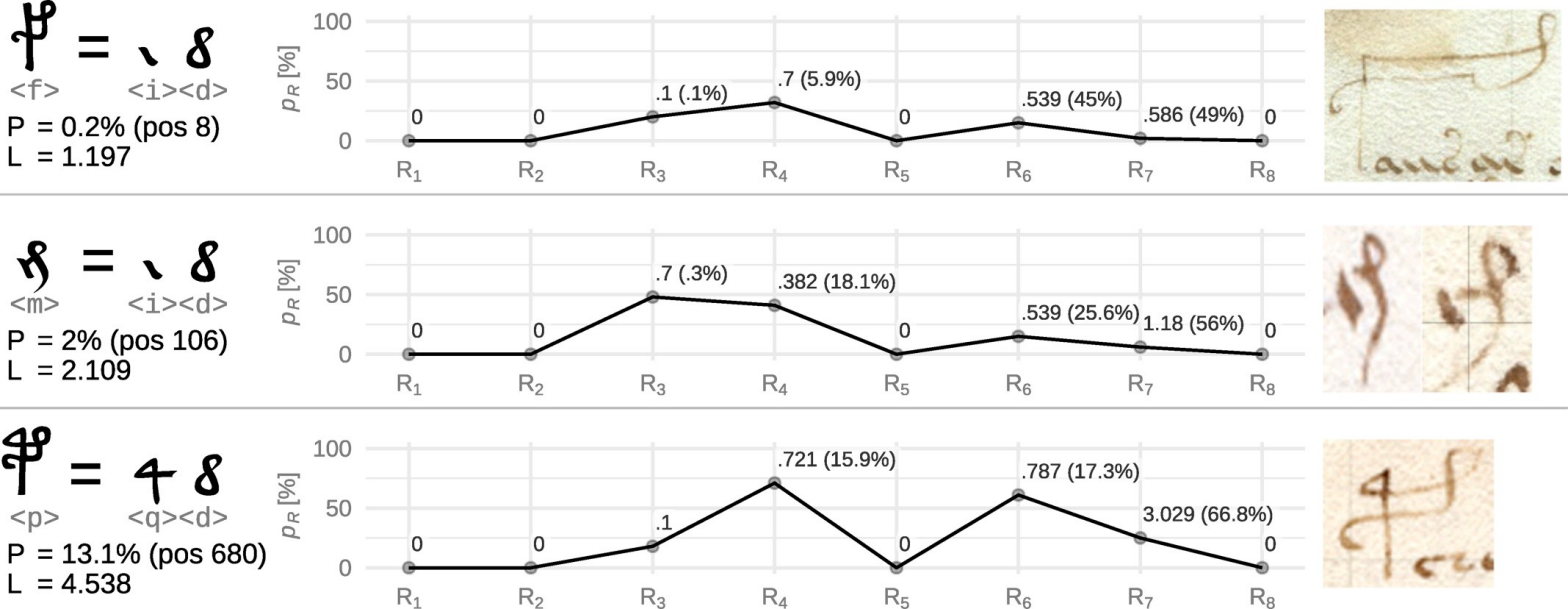
Loss scores of ligature candidates <f>, <m>, and <p>. *P* stands for the percentage of finding better candidate triplet by its loss score, *pos* stands for the rank of the loss score, *L* for the overall triplet loss score ℒ_*T*_. Individual rules R are plotted at axis *x*, probability of finding a better loss score for the given rule among any other triplets, *p*_*R*_ is plotted at axis *y*. Point label contains weighted rule loss *R*_*n*_ and overall loss contribution of the rule in the brackets. Illustrative examples of the symbols were taken from (from left-to-right, up-to-bottom): f20v, f108v, f34v, f4v.

The symbol that acquired the best *ligatureness* score is <f>. Among the possibilities, we gradually find: <fio>, <fie>, <fih>, <fiy>, <flh>, <fia>, <fic>, and finally <fid>, which, as we believe, fits the overall shape, and whose quantitative properties are pointing to its theoretical credibility, see Fig 6 for overall and individual rule loss scores.

Another notable part of this symbol is its prominent curved “c” *ending* (if the symbol is written from the bottom-up) which may be an embellishment, but, as we may notice from an example at folio 20v, we may suspect that this curve could be in fact a third symbol, specifically the symbol <e>. The same idea of an ending <e> then applies for the symbol <p> that we examine later. Also, Currier [23] pointed out that the symbols <f> and <p> are never followed by <e> (even though it is one of the most frequent symbols), which might be explained by its *participation* in the <f> symbol. In such a case, this symbol may not be just a ligature of two symbols, but of three: <ide>. It is also remarkable that finding these graphically appealing component candidates amongst the overall best scoring variants may be possibly viewed as an affirmation of the method working in similar way as for Latin and Arabic described before.

### Ligature candidate <m>

The second-best *ligatureness* score was acquired by <m>. From the ordered list, we greedily selected <m id> as the best explainable combination (see S3 Fig for the visual comparison). Its quantitative properties also reveal its overall great performance, see Fig 6 for overall and individual loss scores.

This proposed triplet shares the same or a better score with only 2.038% of all other triplets, supporting its plausibility. Examining the manuscript, we find examples that stress the role of <i> and the second symbol, assumably symbol <d>. Another remarkable property of <m> is that it is used as the last symbol of 1,061 words and 55× elsewhere. We may assume that <m> is a substitute of symbols <id> dedicated for word endings (a case that explicitly occurs just twice in the manuscript, regarding the transliteration, however one such occurrence is highly doubtable, see S2 Fig and <id> transition). The other visually appealing possibility of <il> is placed at rank 237.

### Ligature candidate <p>

In analogy with the previously discussed symbol <f>, we may consider symbols <qd> as the components of our next ligature candidate <p>, see S3 Fig for the visual comparison. However, by looking into Table 5, we find that the symbol <q> could be a ligature itself (see the following section). We thus find ourselves in a situation where <p> could be a ligature of even three or four symbols. The first two symbols are presumably comprised in the symbol <q> and the other symbols might be <d> and (as discussed above) possibly <e>. Let us assume symbol <p> as a ligature of only the two symbols <qd> as the designed method does not cope with more than two symbols, see Fig 6 loss score details.

The overall loss score ℒ of the triplets does not perform well in comparison to the previous candidates–nearly 13% of the other triplets have the same or even a better score. To understand *why* this visually suitable triplet might have failed, let us inspect the individual loss contributions. The largest contributions are shared by three rules: R7 with ~67% contribution, R6 with ~17%, and R4 with ~16% contribution.

The 7^th^ rule quantifies the idea that a ligature should be used equally or less than it is probable for its two components to meet by random. The resulting loss score of <p> then signalizes that it is used far more frequently than it should be—in an infinitely long random text; however, as has already been discussed, when it comes to non-random texts, this rule might be too strict. The 6^th^ rule quantifies a mismatch in forming a ligature of two symbols out of which at least one should be frequent. The 4^th^ rule then quantifies a mismatch between the left contexts of <p> and <q>. Its high score might have been caused simply by the fact that <q> tends to take quite a specific position within a word—in 5,389 cases it is used as the first symbol and just in 43 cases it is used elsewhere. The opportunities to left-match with the same number of symbols as <p> are then unequal, making the actual loss score possibly smaller.

### Ligature candidate <q>

**Fig 7:**
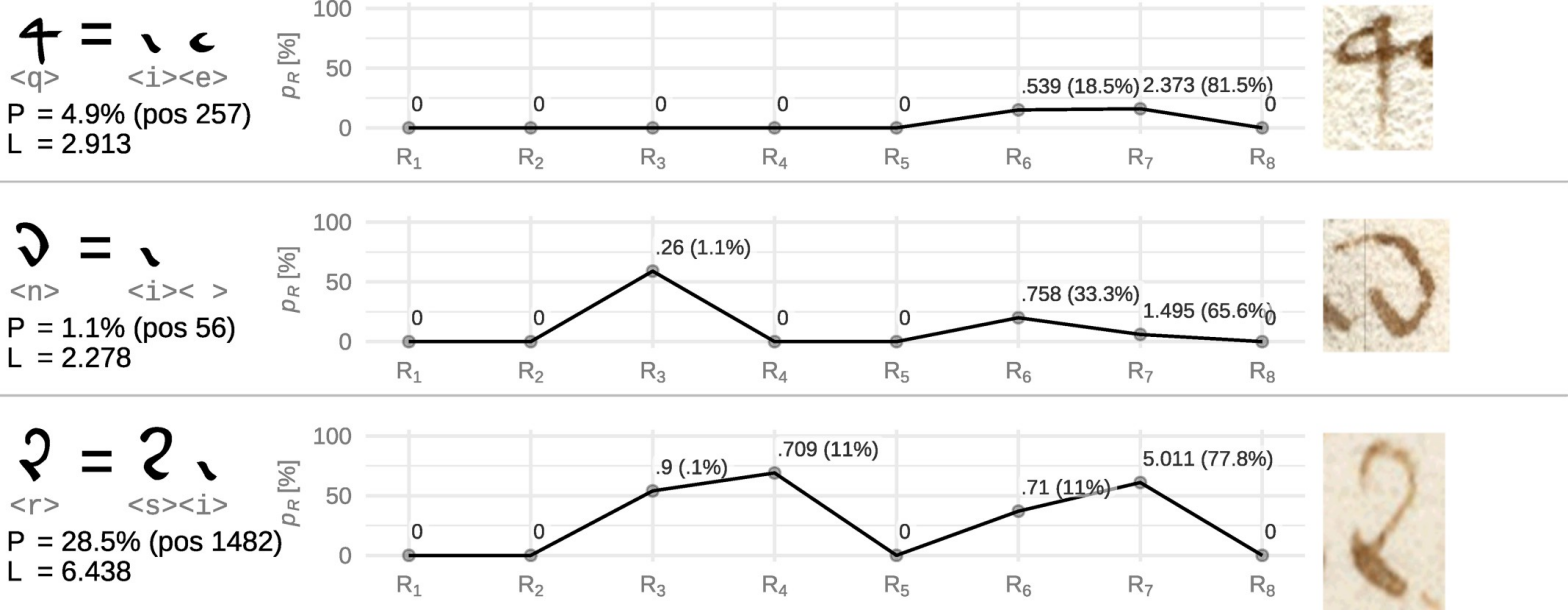
Loss scores of ligature candidates <q>, <n>, <r>. See Fig 6 for details. Symbol illustrations were taken from: f13v, f30v, f27v.

The following ligature candidate is symbol <q>, which, as we observed, could be part of some previous ligature candidates. Looking for its visually plausible components, we find <i, e>, where, in analogy with the previous ligature candidates, the <i> symbol is straightened and raised upwards, while <e> is added on its top (see S3 Fig). The overall performance of this guess is also reasonable, see Fig 7 for the loss scores.

The highest failing rule for this guess is–again–rule 7, contributing ~82% to the overall loss. As discussed previously, this rule signalizes that <q> is used more often than it theoretically should w.r.t. its candidate components in an infinite random text, signalizing structural bias preferring <i> and <e> placed in an immediate succession. Nevertheless, its 4.94^th^ percentile score is reasonable enough to consider <q> as a ligature and thus, probably assume any other symbols that contain it to comprise at least three symbols.

### Ligature candidate <n>

The visual nature of <n>, especially its simplicity in comparison with the previous candidates, hints that something different is going on. Examination of the list of the other offered components does not reveal any satisfactory fit(s) (as in the previous cases), yet we find there an interesting, visually appealing pattern. Candidates placing <i> at the first position tend to have better loss scores than any other ones (measured by loss medians for each starting symbol; the next best scoring median is found with <y> and then it drops in a sudden step). We may thus assume that <i> is the first possible component. The second component then becomes clearer by a peripheral analysis of the typical position of <n>.

In 6,064 cases, <n> is used at the last position within a word, in 77 cases it is placed elsewhere (36× as the pre-final symbol). At the same time, the proposed first component <i>, which is one of the most frequent symbols in the manuscript with 11,650 occurrences, appears at the word ends in just 6 cases (where 1 is doubtable due to possibly mistaken space, see S2 Fig). Therefore, we may suggest that <n> is an embellished way to write <i> on word endings (thus forming a ligature of <i> and [space]). The 6 occurrences of <i> at word endings could be caused by forgetting to embellish the symbol and the 36 pre-final occurrences can be explained by impetuous, mistaken word endings. If we consider *space* as a common symbol and assess the loss score of the proposed triplet, the results show a great performance, see Fig 7 for the details.

If such an assumption is true and the uses of <n> in the middle of words are impetuous, more implications may appear: Why would scribers break the ligature-writing rule instead of making a space and writing the next symbol as the beginning of the next word? At least two explanations emerge: First, breaking letter-to-space order causes grammar or ruleset problems (be it syntactical, semantical, or e.g. word-visual nature); second, the scribers were just reproducing pre-written text and mistook <i> for <n> in the middle of a word.

### Ligature candidate <r>

The next ligature candidate is symbol <r>. When assessing its possible components, we greedily arrive to a pair <si>, see S3 Fig for visual comparison. The loss score of this visually appealing triplet is, however, high, see Fig 7.

The largest portion of the loss score is caused by rule 7, which was already discussed several times above. According to this rule, the ligature appears more often than it should in a truly random text assumption. In texts containing any kind of structure, this assumed ligature would mean that the symbols <s> and <i> tend to be written in succession. By manually checking incidences of <si> in the manuscript, we find there are only two occurrences, both of which could be mistaken by transliteration (see S2 Fig, section <s, i>); this is another example where ligature assumptions point to possible mistakes in transliteration adding the method some more trustfulness. Also, since we assume <n> is the word-end form of <i>, we do not expect to observe a succession of <sn> in the manuscript as we assume it should form the <r> ligature (which is at word endings in ~76% of cases). The <sn> succession has just one occurrence in the whole manuscript.

### Ligature candidate <t>

**Fig 8:**
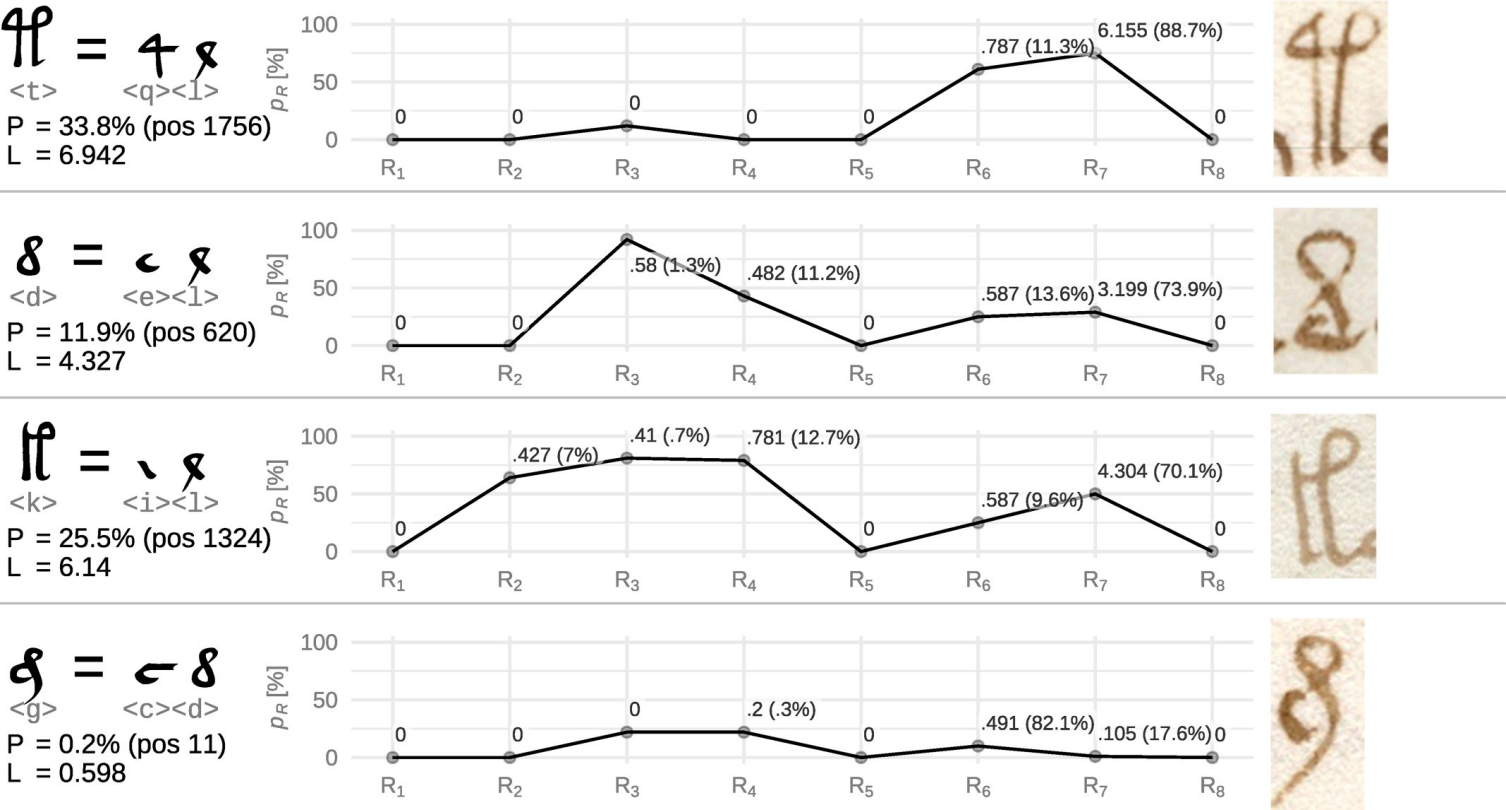
Loss scores of ligature candidates <t>, <d>, <k>, <g>. See Fig 6 for details. Symbol illustrations were taken from: f18v, f2r, f58v, f23v.

The next from the so-called gallows symbols also appears in the potential ligature symbols under the 5^th^ percentile threshold. Examining the shape of this symbol, we find it may be analogous to the previously discussed symbol <p> and thus we may assume the first component to be <q>; the second symbol remains a question. Evaluating the other offered possibilities visually, we come to <ql>, which, in our opinion, meets the essential visual features (see S3 Fig), however, with a quite high loss score, see Fig 8.

Looking further into the loss score of this triplet, we find that it is surprisingly consistent in all rules except rule 7 (contributing ~89% to the loss), which, as discussed before, quantifies estimated probability of its appearance based on a truly random text assumption. Moreover, this rule could have gotten such a high loss score because of <q>, which might be a ligature itself—that is something the rule is not prepared for.

### Ligature candidate <d>

This proposed ligature candidate <d> is, similarly to the candidate <q>, possibly itself a component of some other assumed ligature symbols. Algorithmically, the best proposed components are <ye>, or rather <ey>, offering a reasonable solution (see S3 Fig for visual comparison). D’Imperio [22] also pointed out that this symbol could be a ligature incorporating <e> and <Å> (or <l>), which seems to be more visually appropriate but has a worse overall score, see Fig 8 for the details.

Even though we may perceive <d> as a common Arabic numeral, we should consider its possible ligature nature. Also, if <d> truly is a ligature, then the symbols <f m p> could be ligatures of up to five symbols (in the case of <p>).

### Other symbols

The other symbols listed in Table 5 exceeded the threshold, but we find there one more gallows symbol <k>, whose ligature nature is implied from our previously gained assumptions; the same applies to <g>, which fell below frequency threshold and was thus omitted from the analysis.

### Ligature candidate <k>

The ligature symbol <k> belongs to the group of self-similar symbols called *gallows*. While the rest of gallows are placed below the 5^th^ percentile threshold and show peculiar ACF behavior, <k> resides at 7.459^th^ percentile and does not show any ACF peculiarities. At the same time, we may consider this symbol as the simplest example of the gallows symbols and as such, it does not possess strong ligature features. If we consider <k> as a combination of components of already discussed symbols <f> and <t>, we may imply that its constituents could be <il>, yielding quite high loss score, see Fig 8 for the details and S3 Fig for visual matching.

The high loss score is majorly formed by rule 7, contributing ~70% to the overall loss. If <k> is indeed a ligature of <il>, then <il> would be written more often together than it should be just by random.

### Ligature candidate <g>

The last symbol we discuss is symbol <g>. This symbol is not present in Table 5 as it falls below the 500 occurrence threshold (after a sudden frequency drop), but we can point out some of its similarities with symbol <m>, which we discussed as one of the first ligature candidates. We may assume that <g> may be composed of <d> and some other additional symbol(s). Exploring algorithmically offered variants, we find <cd> as the most visually appealing option (see S3 Fig) yielding also a great loss score, see Fig 8 for the details.

Even though the loss score of this triplet is extremely low, it needs to be stressed that it is based on 94 instances only and thus may be less credible. Aside from the described visual similarity to <m> and their probably shared component symbol, we find another similarity: symbol <g> is also primarily placed at word endings, specifically in 73 cases, while in 4 cases it is placed as the pre-final symbol, in 11 cases as a single symbol and in 8 cases elsewhere.

### Candidates summary

In summary, several of the Voynich symbols may be assumed to be ligatures, especially the symbols <f, m, q, n, g>. When examined as triplets (ligature and two possible components) and even after being paired with their visually plausible components, all of them placed under 5th percentile threshold. The other two gallows symbols–<p> and <t>–were disqualified from the top ligature guesses due to failing rule 7. However, as we found out, rule 7 may be too strict for structured texts. Nevertheless, both these gallows are more complex than the other well-fitting ligature candidate <f>. Concerning rule 7, it could also possibly fail due to the fact that some of the components might be ligatures themselves—consider the case of <p> and <t>, which may comprise <q> or even <d>, making them ligatures of three to five symbols. The ligatures may also explain the ACF misbehavior, as we discuss below.

## Discussion

If we assume that the above-discussed symbols are indeed ligatures, it could have important consequences for the research of the manuscript. First, the alphabet of the manuscript would shrink from 19 typically used EVA symbols (with frequencies higher than 500) to just 10 symbols. The combinatorics of the symbols, words, or lines would change, along with the word lengths, their distributions, context entropy, and other quantitative text properties. The most important change would be, however, the implied small alphabet itself.

What could the Voynich manuscript be if it is indeed realized by such a small alphabet? Ten symbols (or 9 + 1 symbols) may point to a numeric code, which was a common way to conceal information in considered 14^th^ and 15^th^ century–in that time, letters and numbers could be substituted for one another or for newly derived symbols, see survey in [24]. Reeds [25] also noticed and decrypted hidden messages in numerical tables in a book Steganographia by Johannes Trithemius (written ca. 1500). Small alphabet thus should not be out of the scope.

For example, in [26] we find a description of Johannes Trithemius’s numeric steganographic method, which has some appealing properties when viewed more pragmatically. According to the proposed method, each common letter is first substituted by a unique three-digit number using only numbers 1, 2, and 3 (therefore, A is substituted by 111, B by 112, C by 113, …, and Z by 332). The next step is to hide this number in the number of syllables of words: for example, “hello, how are…” encodes a sequence 2–1–-1, which stands for letter J. This method is steganographic; its aim is to hide the presence of a message within a carrier text, which is complicated as the text needs to be quite elaborate and meaningful. But if there is no necessity to conceal the presence of the message itself, as may be the case of an amply illustrated manuscript, instead of creating a meaningful carrier text, one may use a classical substitution cipher to substitute each of the digits (1, 2, 3) by any arbitrary set of characters (some inspiration for this can be found in Trithemius’s Steganographia [27] and the contemporary use of homophony). For example, digit 1 can be substituted by any of the symbols from a set {A, B, C}, digit 2 by {X, Y, Z}, and digit 3 by {J, K, L}. As simple as this may look, just a single letter A–the number 111 –could be rewritten into 3×3×3 = 27 possible variants (AAA, AAB, AAC, …, BCC, CCC); following this idea, the word “hello” could be rewritten into 27×27×27×27×27 = 27^5 = 14,348,907 variants, which is an immense number, considering that the method does not require any special tools or knowledge. The elegant property of this code (except the numbers) is its perfect readability for anyone knowing the digit substitution sets and the letter-number substitution. After a while, reading such a code is not more difficult than reading the Morse code: e.g. reading YZXAKJCCJBXX reduces into reading 222, 133, 113, 122 (standing for “nice”), thus no special or difficult decryption method is needed. Another interesting property of this code is its fragility: misreading just one symbol shifts the whole code, making the result of the text unreadable (until hitting new line, paragraph, or any resetting sequence–which may be related to Currier’s statement that a voynichese line might be a functional entity [23]–or until making another mistake repairing the reading frame). In combination with the probable fact that we do not read the Voynich symbols right, we may easily fail to read the code even when knowing it is present.

Another interesting and important property of this proposed code is that the digit substitutes are picked arbitrarily by coders’ will. This may explain the observed autocorrelations of all the symbols and repeated *words*. First, the autocorrelations might be a consequence of a habit of picking specific substitutes for single digits or for larger blocks–like letters or words: picking the substitutes randomly each time is mentally exhausting, building a habit accelerates the coding work. Once such habit is established, it boosts the frequency of the substitute symbols locally. Such habits may also naturally fade off as the coder notices the repetition and builds up new habits. Such habits may also explain the proposed *languages* A and B, as the two main coders could encode the text by their own habits before the text was rewritten by individual scribers [23]. The resulting text could look like a pseudorandom text with many autocorrelated properties.

The use of the discussed ligatures would be senseful as they could shorten the three-symbol-based code. Such an idea also has some important implications. First, the usage of ligatures would stem from the underlying encoded text and thus should appear rather randomly–which we observed from the chaotical ACF behavior. Second, if some of the proposed ligatures are comprised of three symbols and if the coders built typical ways of substituting specific letters for them, some of the ligatures could be directly equivalent to some letters, making possible that some of the translation attempts, such as Janick–Tucker [3], Bax [28] could have indeed revealed some letters correctly.

The possible presence of ligatures complicates the reading or even attack attempts on such a code if the key or the ligatures are not known to the reader–contrary to its simplicity when both are known to the reader. Misinterpretation of a single ligature may shift the code and make its content unreadable. The same applies e.g. for line endings or uncertain spaces. Even if these can be read flawlessly, a specific attack must be carried to unravel the digit substituents–presumably with assumptions on underlying natural language. After a certain identification of the substituents, the whole collapses into a substitution cipher, while, again, attacking uncertain language that could be abbreviated or in an uncodified form worsens the outlook on immediate or simple cracking the code.

The last point we wish to address is the roles of the *space*. First, the ACF behavior of *space* is significantly autocorrelated up to 44 tested lags–as the other symbols are. This means that the next word length depends on the previous word length and the previous one depends on the one before that etc. If we assume that the autocorrelation is a manifestation of a habit or a free-will choice, then the spaces (and thus the word lengths) may be placed arbitrarily. Therefore, a space may not signalize words, but the visual/psychological desire of the scriber to end a sequence, to mimic words or to evade specific symbol follow up. Currier [23] also noted the word endings affect the beginnings of the following words in Language B which he considers to be unique–if the spaces are an arbitrary choice, they could separate the whole consistent words into its parts and thus possibly explain this phenomenon. A critical role is, however, assumed for <o>.

Finally, preliminary results based on simulations show that the proposed steganographic code may encode a sample of an English fiction and produce multiple quantitative indicators as the Voynich Manuscript.

## Conclusions

This article showed that the Voynich Manuscript is truly distinctive to 21 tested natural language text samples based on a property unusual for any natural language text–the autocorrelation of symbol reuse. The manuscript is also truly distinctive from encrypted text samples. The nearest text sample is a pseudorandom text generated by auto-citation method provided by Timm and Schinner software [20].

Most of the Voynich symbols can be considered as significantly autocorrelated for all the tested lags: such symbols *wait* for their next reappearance in dependence on how long they *waited* previously. This significant behavior implies non-rejectable existence of a system that placed these symbols onto their specific positions. On the contrary, we can also find a few symbols that behave chaotically, implying they were possibly ignored by the assumed system or that they were added after the majority of the symbols were placed into the text. The chaotically behaving symbols, except the simplest symbol <i>, primarily belong to a group of graphically complex symbols known as gallows. The assumption that the chaotical symbols could have been added after the others along with the visual features of the gallows symbols, aroused suspicion that they may be ligatures (or *compounds*) of the simpler and systematically placed symbols. In order to assess this idea, a formal method for calculating ligature score for all symbols and all their possible components was introduced.

Using this method, which we tested and tweaked on Latin and Arabic texts and validated on Armenian, English, and Czech texts, we identified the previously discussed symbols as possible ligatures along with their visually plausible components. These assumed ligatures also hold for the whole manuscript–both the language A and B. We may thus assume that many of the symbols are, in fact, ligatures (or compounds) of the other symbols, implicating possible reduction of the typically used Voynich alphabet from 19 to 10 symbols. Such a reduction, along with the detected unique symbol property of autocorrelation, possibly rejects the plain text theory. It is also notable that the assumptions about the ligature nature of some of the symbols did help us find mistakes in Takeshi Takahashi’s transliteration as the mistakes caused peculiar inconsistencies in the score calculations. Finding mistakes in transliteration by assumptions made on hypothesized manuscript rules may point to the possible correctness of such rules. However, the method could produce false positive results–the assumed ligatures should therefore be assessed more thoroughly.

Such findings led us to formulate a hypothetical explanation of the revealed unique properties based on a slightly modified Trithemius steganographic and numeric code (explicated and disclosed ca. 1500). The proposed steganographic or encoding method may explain observed autocorrelations, symbol and word repetitions and possibly presence of languages A and B, lines as functional entities–as described by Currier [23], self-similar words, and others. A text encoded by this method is also easily readable (like the Morse code–when the key is known) and does not demand any decryption routine. Texts encoded by this method are also complicated to attack if all symbols are not read right and the underlying language is of unknown character. Possible presence of ligatures, whose components are uncertain, complicates attacks.

## Supporting information

S1 Fig The original frequency incidence matrix. Rows represent the beginning symbol; columns the following symbols–read from left to right. The red background (with the original frequency) signalize zeroed incidences by 0.2% threshold. The need to check the validity of the incidence matrix appeared right after assessing quality of the algorithm on the Voynich Manuscript, primarily on a ligature candidate <m> that we were quite sure about and which was scoring badly. Checking the non-zero loss scores led us to verify suspiciously low frequency symbol transitions recorded in the matrix M directly inside the manuscript which then led us to reveal the transliteration and the transitions were mostly wrong. Prior assumptions on symbol <m> thus led us to reveal mistakes in the transliteration itself. Paradoxically, the somewhat problematical transliteration then served as blind cross-validation for the ligature assumptions: when the assumed ligature scored badly in a single score and was visually plausible, the assessment showed the reason is a mistake in the transliteration, not in the rules. In other words, transliteration mistakes were found due to assumptions made in the article not by prior checking the original.
(TIFF)

S2 Fig Incidence matrix low frequency assessment. (TIFF)

S3 Fig List of ligature candidates and their visually examined components–the last pair is the accepted on. (TIFF)

S1 Table List of language samples. When appropriate, headers, footers or inserted texts were ignored for purpose of the analysis.
(DOCX)
