# Supplementary figures and images for: The Voynich manuscript: Symbol roles revisited
Source: PLoS One. 2022 Jan 27;17(1):e0260948. doi: 10.1371/journal.pone.0260948 (PMC8794160; doi:10.1371/journal.pone.0260948)

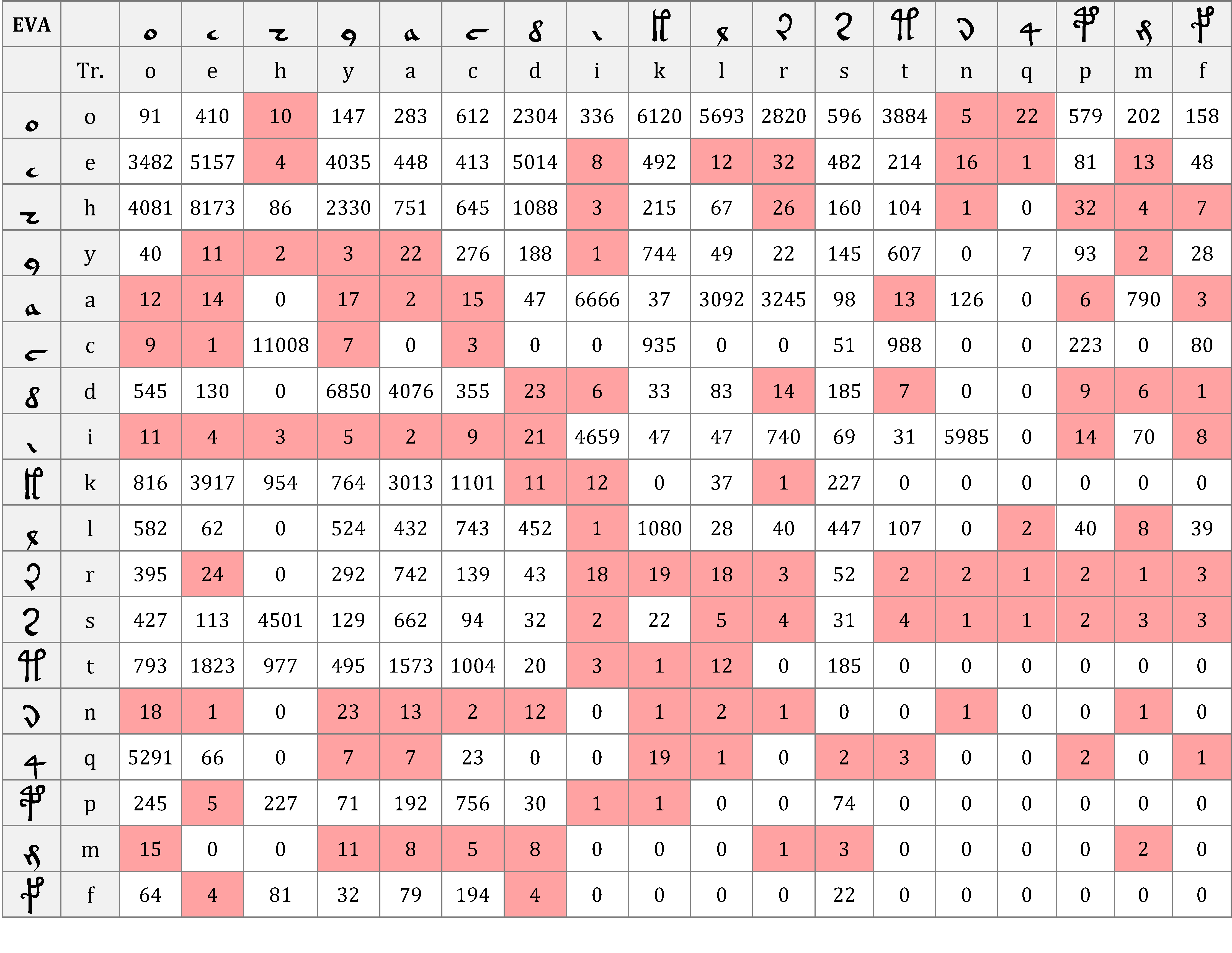

Supplement: S1 Fig — Rows represent the beginning symbol; columns the following symbols–read from left to right. The red background (with the original frequency) signalize zeroed incidences by 0.2% threshold. The need to check the validity of the incidence matrix appeared right after assessing quality of the algorithm on the Voynich Manuscript, primarily on a ligature candidate that we were quite sure about and which was scoring badly. Checking the non-zero loss scores led us to verify suspiciously low frequency symbol transitions recorded in the matrix M directly inside the manuscript which then led us to reveal the transliteration and the transitions were mostly wrong. Prior assumptions on symbol thus led us to reveal mistakes in the transliteration itself. Paradoxically, the somewhat problematical transliteration then served as blind cross-validation for the ligature assumptions: when the assumed ligature scored badly in a single score and was visually plausible, the assessment showed the reason is a mistake in the transliteration, not in the rules. In other words, transliteration mistakes were found due to assumptions made in the article not by prior checking the original. (TIFF) [file pone.0260948.s001.tiff]

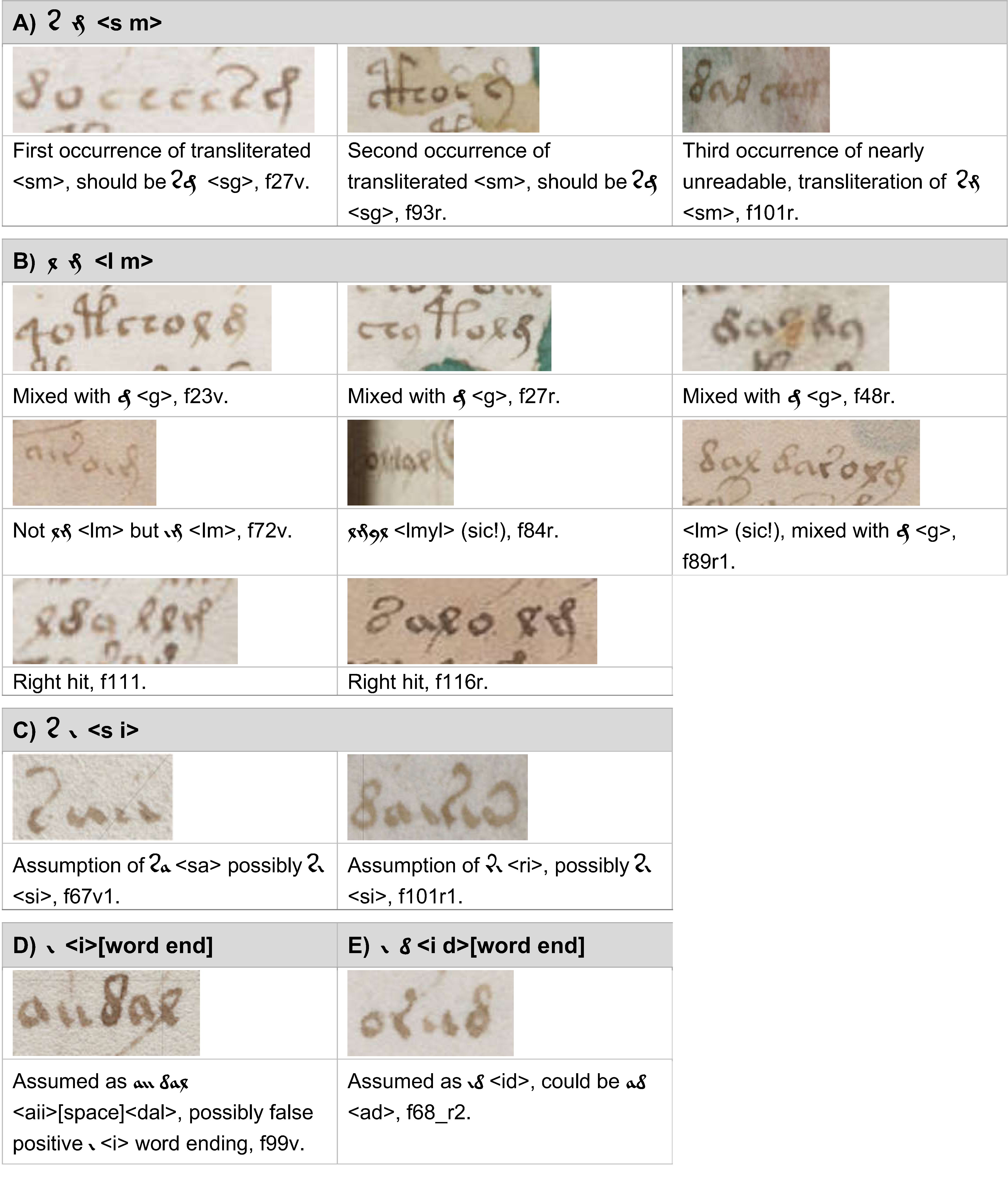

Supplement: S2 Fig — (TIFF) [file pone.0260948.s002.tiff]

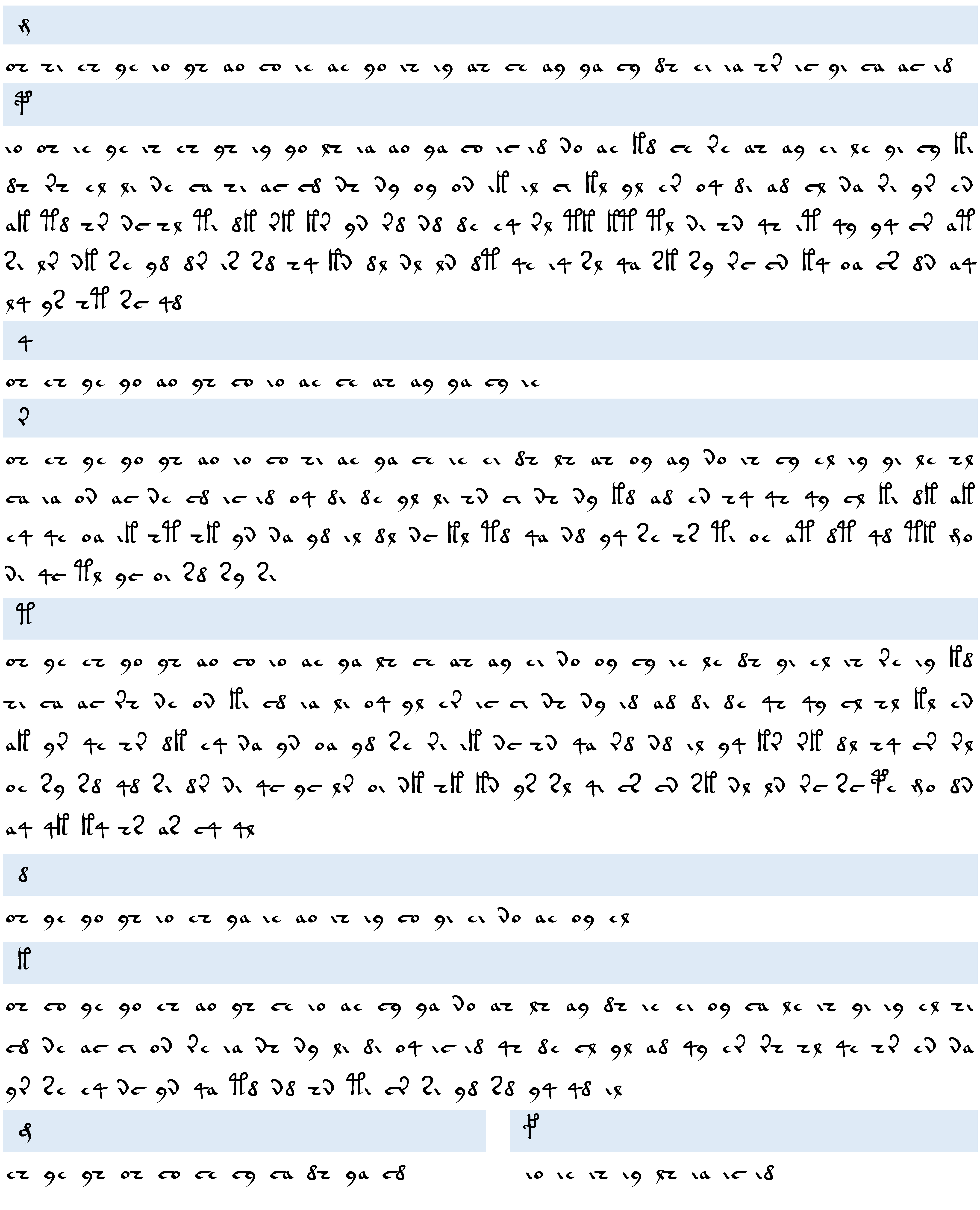

Supplement: S3 Fig — (TIFF) [file pone.0260948.s003.tiff]
