# Supplementary material for: The Voynich manuscript: Symbol roles revisited
Source: PLoS One. 2022 Jan 27;17(1):e0260948. doi: 10.1371/journal.pone.0260948 (PMC8794160; doi:10.1371/journal.pone.0260948)
Supplement: S1 Table — When appropriate, headers, footers or inserted texts were ignored for purpose of the analysis. (DOCX) [file pone.0260948.s004.docx]

| **Language** | **Source** |
| --- | --- |
| Czech | Jaroslav Hašek: Osudy dobrého vojáka Švejka za světové války, online: https://web2.mlp.cz/koweb/00/03/37/00/55/svejk_1_a_2.txt |
| English | George Orwell: Nineteen eighty-four, online: http://gutenberg.net.au/ebooks01/0100021.txt |
| Greek | Grazia Deledda, translated by Christos Alexandridis: Canne al vento, online: https://www.gutenberg.org/files/28658/28658-0.txt |
| Greek (Homer) | Homer, translated by Alexandros Pallis: Illiad, online: https://www.gutenberg.org/files/36248/old/20110529-36248-0.txt |
| Chinese | Ru Zhen Li: Jin Hua Yuan, online: http://archive.org/stream/pgcommunitytexts23818gut/23818-0.txt |
| Italian | Gerolamo Rovetta titulek: Mater dolorosa, online: https://www.gutenberg.org/files/28910/28910-8.txt |
| Italian (Calabrian: Neapolitan) | Giovanni Battista Guarini, translated by Domenico Basile: Il pastor fido in lingua napolitana, online: http://www.gutenberg.org/files/17835/17835-0.txt |
| Latin | Caius Silius Italicus: Punicorum Libri Septemdecim, online: https://www.gutenberg.org/files/27219/27219-0.txt |
| Latin (Virgil) | Virgil: The Aeneid, online: https://www.gutenberg.org/files/227/227.txt |
| Polish | Edward Lasker, translated by Wojciech Ozimiński: Szachy i Warcaby: Droga do mistrzostwa, online: https://www.gutenberg.org/files/15201/15201-8.txt |
| Russian | Leo Tolstoy: War and Peace, online: http://tolstoy.ru/creativity/fiction/1071/ |
| Sanskrit | Rigveda, Mandala, online: https://sanskritdocuments.org/mirrors/rigveda/sanskrit03/RV0301noaccent.html |
| The Bibles | Included both the Old and New Testaments, all versions are available at: bible.com |
| Voynich Data | online: http://kol.ff.upol.cz/voynich_data |
